# Leptin gene variants and colorectal cancer risk: Sex-specific associations

**DOI:** 10.1371/journal.pone.0206519

**Published:** 2018-10-31

**Authors:** Kelsey A. Chun, Jonathan M. Kocarnik, Sheetal S. Hardikar, Jamaica R. Robinson, Sonja I. Berndt, Andrew T. Chan, Jane C. Figueiredo, Noralane M. Lindor, Mingyang Song, Robert E. Schoen, Richard B. Hayes, John D. Potter, Rami Nassir, Stéphane Bézieau, Loic Le Marchand, Martha L. Slattery, Emily White, Ulrike Peters, Polly A. Newcomb

**Affiliations:** 1 Public Health Sciences Division, Fred Hutchinson Cancer Research Center, Seattle, WA, United States of America; 2 Institute of Translational Health Sciences, University of Washington, Seattle, WA, United States of America; 3 Huntsman Cancer Institute, University of Utah, Salt Lake City, UT, United States of America; 4 Division of Cancer Epidemiology & Genetics, National Cancer Institute, Bethesda, MD, United States of America; 5 Division of Gastroenterology, Massachusetts General Hospital and Harvard Medical School, Cambridge, MA, United States of America; 6 Department of Preventive Medicine, University of Southern California, Los Angeles, CA, United States of America; 7 Samuel Oschin Comprehensive Cancer Institute, Cedars-Sinai Medical Center, Los Angeles, CA, United States of America; 8 Department of Health Sciences Research, Mayo Clinic, Scottsdale, AZ, United States of America; 9 Clinical and Translational Epidemiology Unit, Massachusetts General Hospital and Harvard Medical School, Boston, MA, United States of America; 10 Department of Nutrition, Harvard T.H. Chan School of Public Health, Boston, MA, United States of America; 11 Division of Gastroenterology, Hepatology, and Nutrition, University of Pittsburgh, Pittsburgh, PA, United States of America; 12 Division of Epidemiology, Department of Population Health, New York University School of Medicine, New York, NY, United States of America; 13 Department of Epidemiology, University of Washington, Seattle, WA, United States of America; 14 Department of Biochemistry and Molecular Medicine, University of California-Davis, Davis, CA, United States of America; 15 Service de Génétique Médicale, Université de Nantes, Nantes, France; 16 Epidemiology Program, University of Hawai‘i Cancer Center, Honolulu, HI, United States of America; 17 Department of Internal Medicine, University of Utah Health Sciences Center, Salt Lake City, UT, United States of America; Institut d'Investigacions Biomèdiques August Pi i Sunyer (IDIBAPS), SPAIN

## Abstract

**Background:**

High levels of serum leptin and low levels of serum adiponectin are strongly correlated with obesity, a well-established risk factor for colorectal cancer (CRC). Growing evidence suggests that dysregulation of leptin and adiponectin levels may play an etiological role in colorectal carcinogenesis. We evaluated 20 candidate variants in 4 genes previously shown to alter serum leptin and adiponectin levels for associations with obesity (BMI>30 kg/m^2^) and CRC risk.

**Methods:**

We analyzed 6,246 CRC cases and 7,714 population-based controls from 11 studies within the Genetics and Epidemiology of Colorectal Cancer Consortium (GECCO). Associations of each variant with obesity or CRC were evaluated using multivariate logistic regression models stratified by sex and adjusted for age, a study variable, and the first three principal components of genetic ancestry. Gene-specific False Discovery Rate (FDR)-adjusted *p*-values <0.05 denoted statistical significance.

**Results:**

Two variants in the leptin gene showed statistically significant associations with CRC among women: *LEP* rs2167270 (OR = 1.13, 95% CI: 1.06–1.21) and *LEP* rs4731426 (OR = 1.09, 95% CI: 1.02–1.17). These associations remained significant after adjustment for obesity, suggesting that leptin SNPs may influence CRC risk independent of obesity. We observed statistically significant interactions of the leptin variants with hormone replacement therapy (HRT) for CRC risk; these variant associations were strengthened when analyses were restricted to post-menopausal women with low estrogen exposure, as estimated by ‘never use’ of HRT and/or non-obese BMI. No variants were associated with CRC among men.

**Conclusions:**

Leptin gene variants may exhibit sex-specific associations with CRC risk. Endogenous and exogenous estrogen exposure may modify the association between these variants, leptin levels, and CRC risk.

## Introduction

Both high levels of serum leptin and low levels of serum adiponectin are strongly correlated with obesity [1, 2], which is a well-established risk factor for colorectal cancer (CRC) [3]. Whether there is a direct relationship between aberrant leptin and adiponectin levels and CRC independent of obesity, however, is not well characterized. Previous evidence suggests that leptin and adiponectin—two adipocyte-derived hormones that are involved in the regulation of energy homeostasis and insulin sensitivity [4]—may influence the colorectal carcinogenic pathway in opposing ways. Leptin has been shown to act as a growth factor in colonic epithelial cells, promoting tumor cell proliferation and migration and suppressing apoptosis [5]. Adiponectin, by contrast, has been shown to exert an anti-tumorgenic effect by inhibiting cancer cell growth and inducing apoptosis [6].

Epidemiological studies have yielded inconsistent findings on the relationship between adipokine levels and CRC. Several studies have found higher serum leptin [7, 8] and lower serum adiponectin [9–11] levels to be associated with elevated CRC risk, while other studies report conflicting [12–14] or null [15, 16] associations. In addition, several studies have reported that the relationship between adipokine levels and CRC varies by sex [17–21], with stronger positive associations reported in males. Genetic variation in the genes encoding these hormones, or their hormone receptors, may contribute to these differential associations. Greater insight into the role of leptin and adiponectin gene variants in colorectal carcinogenic pathways may therefore contribute to our understanding of the genetic basis of CRC risk.

This study investigated the potential role of candidate gene variants of leptin and adiponectin in sex-specific pathways of obesity and colorectal carcinogenesis. A literature search for variants reported to alter circulating leptin or adiponectin levels identified 20 variants in the genes encoding leptin, adiponectin, and their respective receptors. Associations between these variants with obesity and CRC risk were evaluated using the large data resources of the Genetics and Epidemiology of Colorectal Cancer Consortium (GECCO) [22, 23].

## Materials and methods

### Variant selection

A literature search in the PubMed database identified studies that characterized the association between gene variants in leptin (*LEP*), leptin receptor (*LEPR*), adiponectin (*ADIPOQ*), or adiponectin receptor (*ADIPOR1*) and serum levels of leptin or adiponectin. Variants previously reported as significantly associated with serum leptin or adiponectin levels in at least one prior study were included in our analysis [24–38]. Variants reported as significant predictors of CRC in at least one prior study were included, regardless of whether the gene variants had also been linked to serum hormone levels [39–47]. In total, this literature search identified 20 variants across these four genes: 3 in *LEP*, 3 in *LEPR*, 11 in *ADIPOQ*, and 3 in *ADIPOR1*.

### Study population

This analysis utilized 6,246 CRC cases (4,996 colon cancer, 1,108 rectal cancer) and 7,714 population-based controls pooled from 11 epidemiologic studies within GECCO. This included: the French Association Study Evaluating RISK for sporadic colorectal cancer (ASTERISK); Hawaii Colorectal Cancer Studies 2&3 (Colo2&3); Diet, Activity, and Lifestyle Study (DALS); Health Professionals Follow-up Study (HPFS); Multiethnic Cohort (MEC); Nurses’ Health Study (NHS); Physician's Health Study (PHS); Prostate, Lung, Colorectal Cancer, and Ovarian Cancer Screening Trial (PLCO); Postmenopausal Hormone Study (PMH); VITamins And Lifestyle (VITAL); and Women's Health Initiative (WHI). Study design and inclusion details regarding individual studies within GECCO have been previously described [22, 23]. Colorectal cancer cases were previously defined by GECCO via ICD-9 codes as stage 1–4 colorectal adenocarcinoma. Obesity was defined as body mass index (BMI) ≥ 30 kg/m^2^, consistent with the World Health Organization (WHO) definition [48].

### Genotyping and imputation

We used existing genotype data from prior genome-wide association studies (GWAS) performed in GECCO, the details of which have been previously described [22, 23]. Briefly, genotyping and quality control were performed using a standard process that excluded variants that did not meet quality control measures for Hardy-Weinberg Equilibrium in controls (HWE *p*<10^−4^) or low call rate (<98%). To increase coverage, genotypes were imputed to the Haplotype Reference Consortium (version 1) [49], excluding variants that had a low minor allele count (MAC < 5) or poor imputation accuracy (R^2^ < 0.3).

### Statistical analysis

For each candidate variant, we examined the association with obesity and CRC risk. We calculated odds ratios (ORs) and 95% confidence intervals (95% CI) using multivariate logistic regression, with models stratified by sex and adjusted for age, a study variable that accounts for genotyping platform, and the first three principal components of genetic ancestry [50]. Sensitivity analyses conducted for variant associations with CRC were additionally adjusted for obesity (obese/non-obese BMI). ORs refer to the risk per coded allele. Sex-stratified analyses were conducted based upon previous evidence of the sex-dependent nature of relationships between obesity and CRC risk [51], serum leptin and colorectal adenoma risk [17], and serum adiponectin and CRC risk [18].

To account for multiple comparisons, False Discovery Rate (FDR)-adjusted *p*-values were calculated for variants within sex and within each gene or receptor, with *p*_*FDR*_ <0.05 considered statistically significant [52]. Wald tests evaluated potential gene-gene and gene-environment interactions. All statistical tests and *p*-values were two-sided. All analyses were performed using STATA version 14.0 (StataCorp, College Station, TX).

### Ethics statement

This study was approved by the Institutional Review Board at the Fred Hutchinson Cancer Research Center, Seattle, WA, USA.

## Results

Selected characteristics of the studies and participants included in our analysis are described in Tables 1 and S1. The mean age of study participants across 11 studies was 63.8 years old, and mean BMI was 26.9 kg/m^2^.

**Table 1:** Demographic and baseline characteristics of colorectal cancer cases and controls.

	Cases N = 6,246		Controls N = 7,714	
	Women N = 3,591 (%)	Men N = 2,655 (%)	Women N = 4,643 (%)	Men N = 3,071 (%)
**Age, years**				
Under 50	112 (3)	172 (6)	275 (6)	226 (7)
50–59	728 (20)	559 (21)	1,203 (26)	853 (28)
60–69	1,635 (46)	1,106 (42)	2,150 (46)	1,272 (41)
70–79	1,047 (29)	761 (29)	980 (21)	678 (22)
80+	69 (2)	57 (2)	35 (1)	42 (1)
**BMI, kg/m**^**2**^ [48]*				
Underweight, <18.5	31 (1)	7 (0)	55 (1)	8 (0)
Normal, 18.5–24.9	1,192 (33)	654 (25)	1,855 (40)	979 (32)
Overweight, 25.0–29.9	1,105 (31)	984 (37)	1,387 (30)	1,158 (38)
Obese Class I, 30.0–34.9	561 (16)	330 (12)	584 (13)	287 (9)
Obese Class II, 35.0–39.9	204 (6)	79 (3)	172 (4)	71 (2)
Obese Class III, ≥ 40.0	112 (3)	30 (1)	92 (2)	11 (0)
**Postmenopausal**				
Yes	3,419 (95)	—	4,246 (92)	—
No	151 (4)	—	385 (8)	—
HRT use at baseline*		—		—
Yes	1,119 (31)	—	1,870 (48)	—
No	1,916 (53)	—	2,054 (52)	—
EO HRT use at baseline*		—		—
Yes	470 (13)	—	941 (20)	—
No	1,680 (47)	—	2,117 (46)	—
Duration of EO HRT use*		—		—
0 years (Never users)	211 (6)	—	787 (17)	—
>10 years	446 (12)	—	684 (15)	—
≥10 years	270 (8)	—	464 (10)	—
Stage at Diagnosis*				
Stage 1 or local	1,127 (27)	800 (27)	—	—
Stage 2/3 or regional	1,849 (45)	1,301 (44)	—	—
Stage 4 or distant	420 (10)	274 (9)	—	—
Tumor Site*			—	—
Colon	2,863 (80)	2,098 (79)	—	—
Rectum	599 (17)	501 (19)	—	—

BMI = Body Mass Index; HRT = Hormone Replacement Therapy; EO = Estrogen-Only

*Do not sum to total due to missing values

Table 2 describes the candidate SNPs in leptin, adiponectin, and their receptors and summarizes their associations with CRC risk. Two variants in leptin were positively associated with CRC risk among women: *LEP* rs2167270 (OR = 1.13, 95% CI: 1.06–1.21, *p*_*FDR*_ = 0.003) and *LEP* rs4731426 (OR = 1.09, 95% CI: 1.02–1.17, *p*_*FDR*_ = 0.005). None of the 20 candidate variants, however, was statistically significantly associated with CRC risk among men. Testing the interaction between these variants and sex initially suggested a difference in CRC risk by sex for *LEP* variant rs4731426 (*p* = 0.06) but was not significant after FDR adjustment (*p*_*FDR*_ = 0.18). Testing for variant associations under dominant or recessive genetic models did not substantially alter our results.

**Table 2:** Associations of candidate genetic variants with colorectal cancer.

				Women		Men	
Gene	Variant	CA/RA	CAF	OR^*^ (95% CI)	*p*	OR^*^ (95% CI)	*p*
*LEP*	rs2167270	G/A	0.63	1.13 (1.06–1.21)	<0.001^+^	0.96 (0.89–1.04)	0.36
	rs7799039	A/G	0.45	1.06 (0.99–1.13)	0.08	0.99 (0.92–1.07)	0.86
	rs4731426	C/G	0.56	1.09 (1.02–1.17)	0.008^+^	0.99 (0.92–1.07)	0.88
*LEPR*	rs1137101	G/A	0.45	0.99 (0.92–1.05)	0.66	0.98 (0.91–1.06)	0.66
	rs6588147	A/G	0.68	1.01 (0.94–1.08)	0.78	1.04 (0.96–1.13)	0.35
	rs1137100	G/A	0.26	0.97 (0.90–1.04)	0.37	1.00 (0.91–1.09)	0.99
*ADIPOR1*	rs1342387	C/T	0.54	0.99 (0.93–1.06)	0.82	0.99 (0.91–1.06)	0.72
	rs12733285	T/C	0.31	1.03 (0.96–1.11)	0.47	0.98 (0.90–1.07)	0.69
	rs7539542	C/G	0.69	0.98 (0.91–1.06)	0.67	1.00 (0.92–1.09)	0.98
*ADIPOQ*	rs1501299	T/G	0.28	0.96 (0.89–1.03)	0.23	1.00 (0.92–1.09)	0.95
	rs17366743	C/T	0.03	1.00 (0.81–1.25)	0.96	1.05 (0.82–1.35)	0.70
	rs16861194	G/A	0.08	0.99 (0.87–1.13)	0.90	0.95 (0.82–1.10)	0.47
	rs2241766	C/T	0.11	0.97 (0.87–1.07)	0.55	1.01 (0.89–1.13)	0.93
	rs17300539	T/C	0.09	0.86 (0.76–0.98)	0.03	0.99 (0.85–1.14)	0.85
	rs822387	C/G	0.09	0.90 (0.79–1.02)	0.11	1.00 (0.86–1.15)	0.97
	rs12495941	T/G	0.35	0.98 (0.91–1.05)	0.56	1.01 (0.93–1.10)	0.77
	rs182052	A/G	0.34	0.98 (0.91–1.05)	0.51	0.96 (0.88–1.04)	0.35
	rs822396	A/G	0.81	0.96 (0.88–1.04)	0.34	1.02 (0.92–1.12)	0.72
	rs822395	A/C	0.65	1.00 (0.93–1.07)	0.94	1.03 (0.95–1.12)	0.47
	rs1063538	C/T	0.61	1.06 (0.99–1.13)	0.10	1.00 (0.92–1.08)	0.95

CA = Coded Allele; RA = Reference Allele; CAF = Coded Allele Frequency; OR = Odds Ratio per coded allele; CI = Confidence Interval

^*^ Adjusted for age, study, and first three principal components of genetic ancestry

^*+*^ p<0.05 after FDR-adjustment

The two leptin gene variants associated with CRC risk in women were not concomitantly associated with obesity (*p* = 1.00). To assess whether obesity was confounding the association between leptin variants and CRC in women, we adjusted our initial logistic regression model for obesity (obese/non-obese BMI). The associations of *LEP* variants rs2167270 and rs4731426 with CRC risk remained statistically significant after this adjustment (*p*_*FDR*_ ≤ 0.03).

Based on prior studies of sex hormones in relation to leptin levels, as well as CRC risk, we performed stratified analyses according to exogenous estrogen exposure and assessed potential gene-environment interactions between the leptin variants and estrogen exposure with regards to CRC risk among women.

Among post-menopausal women, we observed that the associations of *LEP* rs2167270 and rs4731426 with CRC varied according to history of estrogen-only (EO) hormone replacement therapy (HRT) use, categorically defined as 0 years (Never users), <10 years, and ≥10 years (*p*_*FDR*_ for interaction: 0.02 and 0.02, respectively). We then conducted stratified analyses in post-menopausal women according to history of EO HRT (Table 3). The associations of *LEP* rs2167270 and rs4731426 were non-significant in both categories of EO HRT ever users. In never users, however, the variants were statistically significantly associated with CRC risk, and their effect sizes were strengthened (*LEP* rs2167270: OR = 1.54, 95% CI: 1.19–1.99, *p*_*FDR*_ = 0.003; *LEP* rs4731426: OR = 1.37, 95% CI: 1.08–1.74, *p*_*FDR*_ = 0.03) (*p*_*FDR*_ for interaction: 0.02 and 0.02, respectively).

**Table 3:** Associations of candidate leptin variants with colorectal cancer among post-menopausal women, by duration of estrogen-only hormone replacement therapy use.

		≥10 years (N = 696)		<10 years (N = 998)		0 years (N = 685)	
Gene	Variant	OR^*^ (95% CI)	*p*	OR^*^ (95% CI)	*p*	OR^*^ (95% CI)	*p*
*LEP*	rs2167270	0.97 (0.76–1.23)	0.79	1.06 (0.88–1.29)	0.53	1.54 (1.19–1.99)	0.001^*+*^
	rs7799039	0.81 (0.65–1.02)	0.07	1.05 (0.88–1.26)	0.58	1.37 (1.08–1.74)	0.01^*+*^
	rs4731426	0.96 (0.76–1.20)	0.70	1.01 (0.84–1.22)	0.89	1.37 (1.08–1.74)	0.01^*+*^

EO = Estrogen-Only; HRT = Hormone Replacement Therapy; OR = Odds Ratio per coded allele; CI = Confidence Interval

^*^ Adjusted for age, study, and first three principal components of genetic ancestry

^*+*^ p<0.05 after FDR-adjustment

Given that the associations of leptin variants with CRC were strengthened when analyses were restricted to women with lower exogenous estrogen exposure, as estimated by never EO HRT use, we attempted to assess whether the trend would persist in women with lower endogenous estrogen exposure, using menopausal status and obesity status as proxies. Although no statistically significant interaction effects were observed between the two leptin gene variants and menopausal status or obesity status, in analyses stratified by menopausal status, associations of leptin variants with CRC risk remained statistically significant only among post-menopausal women. In analyses of post-menopausal women stratified by obese versus non-obese BMI, associations of the two leptin variants with CRC remained statistically significant in non-obese women only (Table 4). Among men and pre-menopausal women, analyses stratified by obese versus non-obese BMI did not yield any statistically significant SNP associations.

**Table 4:** Associations of candidate leptin variants with colorectal cancer among post-menopausal women, by obesity status.

		**Obese** **(N = 1,599)**		**Non-obese** **(N = 4,886)**	
**Gene**	**Variant**	OR^*^ **(95% CI)**	***p***	OR^*^ **(95% CI)**	***p***
*LEP*	rs2167270	1.00 (0.86–1.16)	0.99	1.18 (1.08–1.29)	<0.001^*+*^
	rs7799039	0.97 (0.84–1.12)	0.69	1.06 (0.98–1.15)	0.15
	rs4731426	0.96 (0.83–1.11)	0.60	1.13 (1.04–1.23)	0.003^*+*^

OR = Odds Ratio per coded allele; CI = Confidence Interval

^*^ Adjusted for age, study, and first three principal components of genetic ancestry

^*+*^ p<0.05 after FDR-adjustment

Consistent with these observations, the CRC risk conferred by *LEP* variants rs2167270 and rs4731426 was highest among women who fulfilled all three categories of low estrogen exposure: non-obese, post-menopausal, never users of EO HRT (*LEP* rs2167270: OR = 1.69, 95% CI: 1.26–2.26, *p*_*FDR*_ = 0.003; *LEP* rs4731426: OR = 1.49, 95% CI: 1.13–1.96, *p*_*FDR*_ = 0.003). These results suggest that estrogen exposure–both exogenous and endogenous–may modify the association between leptin gene variants and CRC risk.

To examine whether other forms of HRT would have a similar impact on the association of leptin variants with CRC risk, we performed stratified analyses according to estrogen-progesterone (EP) HRT. Unlike with EO HRT use at baseline, associations of leptin variants with CRC risk were statistically significant among both users and non-users of EP HRT at baseline, and the association of both variants with CRC risk was only borderline significant among never users. Overall, EP HRT use did not appear modify SNP effects as strikingly as EO HRT use.

Given evidence in prior studies that obesity is more strongly associated with colon rather than rectal cancer [53], we assessed associations of SNPs with CRC in analyses stratified by subsite. However, no subsite-specific associations were statistically significant in analyses stratified by sex or across the total study population.

We also assessed potential gene-gene interactions between leptin and adiponectin variants with regards to CRC risk. Previous studies have posited that the ratio of circulating leptin to adiponectin levels may be a more salient marker of disease risk than leptin or adiponectin levels alone [19, 54]. Therefore, we hypothesized that the interaction of leptin and adiponectin variants might confer multiplicative CRC risk. However, modeling pairwise interactions between *LEP* and *ADIPOQ* variants for CRC risk did not identify any statistically significant interactions in sex-stratified analyses.

Although not the primary focus on our analyses, we also identified two variants significantly associated with obesity risk (S2 Table). As with the CRC associations, both associations with obesity were sex-specific. *LEPR* rs6588147 was associated with obesity among women (OR = 1.12, 95% CI: 1.03–1.22) but not among men. By contrast, *ADIPOQ* rs17366743 was associated with obesity among men (OR = 0.52, 95% CI: 0.34–0.79) but not among women.

## Discussion

We found evidence for several sex-specific genetic associations with obesity and CRC risk. Two variants in the leptin gene (rs2167270 and rs4731426) were associated with increased CRC risk in women but not in men, and were not concomitantly associated with obesity. These associations remained statistically significant after adjustment for BMI, suggesting that these leptin variants may be associated with colorectal carcinogenesis independently of obesity.

Prior studies have reported associations of intronic variant rs4731426 and exonic variant rs2167270 with circulating leptin levels, suggesting that these SNPs are functional. Dasgupta et al. reported an increasing trend in leptin levels for each addition of the C minor allele in *LEP* rs4731426 [24]. Consistent with this finding, our study observed that the C allele of this variant was associated a modest increased risk of CRC among women.

Prior findings regarding *LEP* rs2167270 in relation to leptin levels and CRC risk have been conflicting. Some studies have observed associations of the A allele of *LEP* rs2167270 –the reference allele in our study–with elevated leptin levels [24, 55]. Contradictory to what we would then expect given the proposed directional effect, several studies have reported significant association of the A allele of this variant with decreased CRC risk [39, 40]. Consistent with these observations, our study observed that the G allele of this variant was associated with a modest increased CRC risk among women.

Linkage disequilibrium analysis of variants rs2167270 and rs4731426 revealed that the G allele and C allele of each variant, respectively, are highly correlated. We therefore matched the reference and coded alleles for *LEP* rs2167270 and rs4731426 to reflect this correlation. In our study, the correlation coefficient between coded alleles of leptin variants rs2167270 and rs4731426 was 0.85. Within NCI LDmatrix (https://analysistools.nci.nih.gov/LDlink/), the variants were found to have an r^2^ value of 0.72.

To our knowledge, ours is the first study to report sex-specific associations between leptin variants and CRC risk among women but not among men. Prior reports of sex differences in the association between adipokine levels and risk for colorectal neoplasia have found leptin and adiponectin levels to be more predictive of risk among men than among women [17–20]. Previously, Chia et al. reported a three-fold increased risk of colorectal adenoma for the highest versus lowest quartile of leptin among men, but found no association among women [17]. Yamaji et al. reported a 40% reduced risk of colorectal adenoma for the highest versus lowest tertile of adiponectin among men, but not among women [19]. Similarly, Song et al. reported a 45% lower risk of CRC for the highest versus lowest quartile of adiponectin among men, but no association among women [18]. Each of these studies’ analyses adjusted for important confounders, including BMI, waist circumference, or some other covariate associated with obesity, and found that their results remained statistically significant. The mechanisms underlying the heterogeneity by sex observed in these studies were unclear, though the authors proposed several hypotheses. One explanation was that sex differences in adipokine levels–in particular, higher levels of both adiponectin and leptin among women compared to men–contributed to the null finding in women [18]. Another explanation was that sex differences in body fat distribution may affect leptin levels and their observed effect on CRC risk [56].

Studies reporting sex differences in the associations between gene variants in leptin and adiponectin and CRC have been similarly limited to findings in men. A study by Partida-Perez et al. found that *LEP* rs2167270 was associated with CRC risk among men but not among women [57]. Slattery et al. reported a sex-specific association in leptin receptor, finding that *LEPR* rs6588147 was associated with increased risk of colon cancer among men but not among women [39].

Our finding on the relationship between leptin variants and CRC risk among women raises new questions regarding the mechanisms by which leptin and leptin gene variants might affect colorectal carcinogenic pathways. Given the interactions we observed between these variants and other factors, we propose mechanisms that may be independent of obesity.

We observed that associations of *LEP* rs2167270 and rs4731426 with CRC risk among women remained significant, and were strengthened, when analyses were restricted to women with low exogenous and endogenous estrogen exposure. The risk conferred by these leptin variants was highest among women who were post-menopausal, non-obese, and had no prior history of EO HRT use. These results suggest that estrogen exposure may modify the association between leptin gene variants and CRC risk. Specifically, estrogen exposure may be protective against the risk conferred by functional leptin variants, such that these variants only exert a detectable effect among women with low lifetime estrogen exposure. The mechanisms underlying this relationship in the context of our study remain unclear. However, prior studies have found suggestive evidence that sex-specific associations between leptin levels and CRC risk may involve functional cross-talk between leptin and estrogen systems [58].

Colorectal cancer incidence is lower among women compared with men, and it has been hypothesized that female hormones may play a protective role [59]. The protective effects of estrogen are believed to be mediated by ERβ –the predominant estrogen receptor in human colon [60]. ERβ expression is markedly and selectively reduced in CRC tissue compared to normal adjacent tissue [60], and inversely associated with stage of colorectal cancer and prognosis [61]. Silencing of ERβ by genetic knockdown or promoter methylation has been previously shown to induce aberrant cell proliferation while upregulation of ERβ has been shown to promote apoptotic signaling, suggestive of ERβ’s role as a tumor suppressor [62].

Leptin, by contrast, has been previously shown to act as a growth factor in colon epithelial cells. Binding of leptin to its receptor activates transcription (JAK/STAT) and Ras/extracellular signal-regulated kinase (ERK1/2) signal transduction pathways [63], inducing tumor cell proliferation and migration and suppressing apoptosis. Interestingly, evidence from prior studies suggests functional crosstalk between estrogen and leptin, with estrogen opposing the oncogenic effects of leptin. In adipocytes, estrogen (E2) administration has been shown to increase ERβ expression and subsequently reduce leptin expression [58]. In hepatic cells, E2 administration has been shown to inhibit leptin-stimulated cell growth (via decreased STAT3 signaling) and enhanced leptin-suppressed apoptosis (via increased SOCS3 signaling) through activation of ERβ [64]. These experimental findings support the results of our study, and taken together, suggest that estrogen exposure may be protective against the oncogenic effects of leptin variants associated with elevated circulating leptin levels.

In addition, that EO but not EP HRT use appeared to be strongly protective against leptin SNP-associated CRC risk in our study aligns with prior evidence for repression of estrogen-stimulated ER activity by liganded progestin receptor [65].

Our results may also shed light on a somewhat controversial hypothesis regarding obesity and CRC in post-menopausal women: that elevated endogenous estrogen in overweight and obese women–specifically postmenopausal women–may attenuate obesity-associated CRC risk [66, 67]. The relationship between obesity and CRC is known to be stronger in men compared in women, and when women are further stratified by menopausal status, the attenuated relationship between obesity and CRC risk is clearly restricted to postmenopausal women [68, 69]. Given the suggested protective effects of estrogen exposure among women and the fact that adipose tissue is the primary source of endogenous estrogen in postmenopausal women, it has been hypothesized that increased adiposity is less pathogenic in postmenopausal women than in their male and premenopausal counterparts. In our study, stratified analyses by obese versus non-obese BMI among postmenopausal women revealed that the CRC risk conferred by *LEP* SNPs was restricted to non-obese women. There was no evidence of leptin-associated CRC risk in the post-menopausal obese women, which may suggest that adiposity in postmenopausal women may confer some protective against the effects of leptin variants or elevated leptin levels.

A key strength of this study is its large sample size, leveraging nearly 14,000 CRC cases and controls pooled from 11 different studies. Although we selected variants that were functionally associated with protein expression, we nonetheless did not directly measure circulating leptin or adiponectin. In addition, our measures for estimating endogenous estrogen using menopausal status and obesity are only approximations. Despite the study’s limitations, we believe the results provide compelling suggestive evidence for the role of estrogen in modifying the carcinogenic effects of leptin. To our knowledge, this is the first study to report on estrogen as an effect modifier of leptin in an epidemiologic setting.

Validation studies evaluating our sex-specific genetic findings for CRC risk with directly measured serum levels of leptin, estrone, and estradiol would be very informative. Future studies should also be directed towards discerning the functional roles of these two leptin variants, which still remain largely unclear. Although candidate SNPs were selected for this study based on previously reported association with leptin levels, more direct laboratory investigation of these variants is still needed. To our knowledge, no experimental studies have adequately assessed the functional role of these variants beyond their correlation with leptin levels.

In conclusion, our study investigated the role of leptin and adiponectin in obesity and colorectal cancer using a candidate gene variant approach. Analyses revealed statistically significant sex-specific associations between two leptin gene variants–*LEP* rs2167270 and *LEP* rs4731426 –and CRC risk among women but not men. These gene variants in leptin were not associated with obesity risk among women and remained significantly associated with CRC after adjustment for obese vs non-obese BMI. In addition, these associations were strengthened when analyses were restricted to women with low estrogen exposure, as estimated by post-menopausal status, never use of hormone replacement therapy, and non-obese BMI. These results suggest that estrogen exposure may modify the association between leptin gene variants and CRC risk.

## Supporting information

S1 Table Characteristics of study participants across 11 studies in the Genetic Epidemiology of Colorectal Cancer Consortium (GECCO). (DOCX)

S2 Table Associations of candidate genetic variants with obesity. (DOCX)
