# Supplementary material for: Leptin gene variants and colorectal cancer risk: Sex-specific associations
Source: PLoS One. 2018 Oct 31;13(10):e0206519. doi: 10.1371/journal.pone.0206519 (PMC6209341; doi:10.1371/journal.pone.0206519)
Supplement: S1 Table — (DOCX) [file pone.0206519.s001.docx]

**S1 Table.** Characteristics of study participants across 11 studies in the Genetic Epidemiology of Colorectal Cancer Consortium (GECCO)

| **Study** | **No. of cases** | **No. of controls** | **% Women** | **Mean age (SE), years** | **Mean BMI (SE), kg/m^2^** |
| --- | --- | --- | --- | --- | --- |
| ASTERISK | 892 | 947 | 42 | 65.2 (0.3) | -- |
| Colo2&3 | 87 | 125 | 45 | 65.2 (0.8) | 26.2 (0.4) |
| DALS | 1,116 | 1,174 | 45 | 63.9 (0.2) | 27.1 (0.1) |
| HPFS | 172 | 575 | 0 | 63.3 (0.3) | 25.6 (0.1) |
| MEC | 328 | 346 | 46 | 63.0 (0.3) | 26.5 (0.2) |
| NHS | 298 | 1,352 | 100 | 58.9 (0.2) | 25.4 (0.1) |
| PHS | 375 | 389 | 0 | 58.9 (0.3) | 25.1 (0.1) |
| PLCO | 987 | 865 | 41 | 64.2 (0.1) | 27.6 (0.1) |
| PMH | 280 | 122 | 100 | 62.8 (0.4) | 27.3 (0.3) |
| VITAL | 280 | 288 | 47 | 66.5 (0.3) | 27.7 (0.2) |
| WHI | 1,431 | 1,531 | 100 | 66.4 (0.1) | 28.0 (0.1) |
| Total | 6,246 | 7,714 | 59 | 63.5 (0.1) | 26.9 (0.0) |
