# Supplementary material for: Leptin gene variants and colorectal cancer risk: Sex-specific associations
Source: PLoS One. 2018 Oct 31;13(10):e0206519. doi: 10.1371/journal.pone.0206519 (PMC6209341; doi:10.1371/journal.pone.0206519)
Supplement: S2 Table — (DOCX) [file pone.0206519.s002.docx]

**S2 Table.** Associations of candidate genetic variants with obesity

|  | |  |  | **Women** | | **Men** | |
| --- | --- | --- | --- | --- | --- | --- | --- |
| **Gene** | **Variant** | **CA/RA** | **CAF** | **OR^*^ (95% CI)** | ***p*** | **OR^*^ (95% CI)** | ***p*** |
| *LEP* | rs2167270 | G/A | 0.63 | 0.98 (0.90-1.06) | 0.55 | 0.99 (0.88-1.11) | 0.86 |
|  | rs7799039 | A/G | 0.45 | 0.99 (0.92-1.07) | 0.84 | 1.05 (0.94-1.17) | 0.40 |
|  | rs4731426 | C/G | 0.56 | 0.98 (0.91-1.06) | 0.63 | 1.05 (0.94-1.18) | 0.38 |
| *LEPR* | rs1137101 | G/A | 0.45 | 0.98 (0.90-1.06) | 0.57 | 1.00 (0.90-1.12) | 0.98 |
|  | rs6588147 | A/G | 0.68 | 1.12 (1.03-1.22) | 0.008^+^ | 0.91 (0.81-1.03) | 0.13 |
|  | rs1137100 | G/A | 0.26 | 0.95 (0.87-1.04) | 0.25 | 1.08 (1.95-1.23) | 0.22 |
| *ADIPOR1* | rs1342387 | C/T | 0.54 | 1.00 (0.92-1.08) | 0.93 | 1.06 (0.95-1.19) | 0.27 |
|  | rs12733285 | T/C | 0.31 | 0.96 (0.88-1.05) | 0.41 | 0.91 (0.81-1.03) | 0.15 |
|  | rs7539542 | C/G | 0.69 | 1.01 (0.92-1.10) | 0.85 | 1.03 (0.91-1.17) | 0.65 |
| *ADIPOQ* | rs1501299 | T/G | 0.28 | 0.98 (0.89-1.07) | 0.59 | 1.01 (0.89-1.14) | 0.90 |
|  | rs17366743 | C/T | 0.03 | 1.22 (0.96-1.56) | 0.10 | 0.52 (0.34-0.79) | 0.002*^+^* |
|  | rs16861194 | G/A | 0.08 | 1.09 (0.94-1.26) | 0.27 | 1.05 (0.85-1.29) | 0.66 |
|  | rs2241766 | C/T | 0.11 | 1.04 (0.92-1.17) | 0.54 | 1.08 (0.91-1.29) | 0.36 |
|  | rs17300539 | T/C | 0.09 | 0.85 (0.73-1.00) | 0.04 | 1.00 (0.81-1.23) | 0.98 |
|  | rs822387 | C/G | 0.09 | 0.83 (0.71-0.97) | 0.02 | 1.02 (0.83-1.25) | 0.88 |
|  | rs12495941 | T/G | 0.35 | 0.99 (0.91-1.08) | 0.85 | 0.97 (0.85-1.09) | 0.56 |
|  | rs182052 | A/G | 0.34 | 1.03 (0.95-1.12) | 0.44 | 0.96 (0.85-1.08) | 0.50 |
|  | rs822396 | A/G | 0.81 | 0.97 (0.88-1.07) | 0.59 | 0.93 (0.80-1.07) | 0.29 |
|  | rs822395 | A/C | 0.65 | 1.05 (0.96-1.14) | 0.30 | 0.92 (0.82-1.04) | 0.20 |
|  | rs1063538 | C/T | 0.61 | 1.01 (0.93-1.09) | 0.84 | 0.97 (0.87-1.08) | 0.55 |

CA = Coded Allele; RA = Reference Allele; CAF = Coded Allele Frequency; OR = Odds Ratio per coded allele; CI = Confidence Interval

*^*^* Adjusted for age, study, and first three principal components of genetic ancestry

*^+^* p<0.05 after FDR-adjustment
